# Supplementary material for: Outcomes of Patients Receiving a Kidney Transplant or Remaining on the Transplant Waiting List at the Epicentre of the COVID-19 Pandemic in Europe: An Observational Comparative Study
Source: Pathogens. 2022 Oct 3;11(10):1144. doi: 10.3390/pathogens11101144 (PMC9610233; doi:10.3390/pathogens11101144)
Supplement: Supplementary file 1 [file pathogens-11-01144-s001.zip › Supplementary Figure S2.pdf]

Supplementary Figure S2. 1-year death-censored kidney allograft survival rate before (Pre-COV-KTR, solid line) or during (COV-KTR, dashed line) the COVID-19 pandemic (Analysis A2).

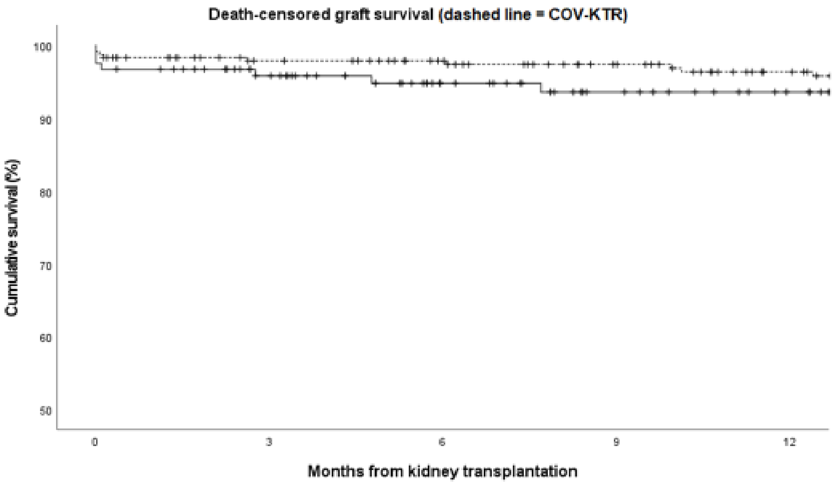

	Baseline (%)	3 months (%)	6 months (%)	12 months (%)
Pre-COV-KTR	100	96	95	93
COV-KTR	100	98	98	96
Number of patients exposed				
Pre-COV-KTR	122	116	94	65
COV-KTR	238	230	212	184

P = 0.282
