# Supplementary material for: Outcomes of Patients Receiving a Kidney Transplant or Remaining on the Transplant Waiting List at the Epicentre of the COVID-19 Pandemic in Europe: An Observational Comparative Study
Source: Pathogens. 2022 Oct 3;11(10):1144. doi: 10.3390/pathogens11101144 (PMC9610233; doi:10.3390/pathogens11101144)
Supplement: Supplementary file 1 [file pathogens-11-01144-s001.zip › Supplementary Figure S3.pdf]

Supplementary Figure S3. 1-year cumulative acute rejection rate before (Pre-COV-KTR, solid line) or during (COV-KTR, dashed line) the COVID-19 pandemic (Analysis A2).

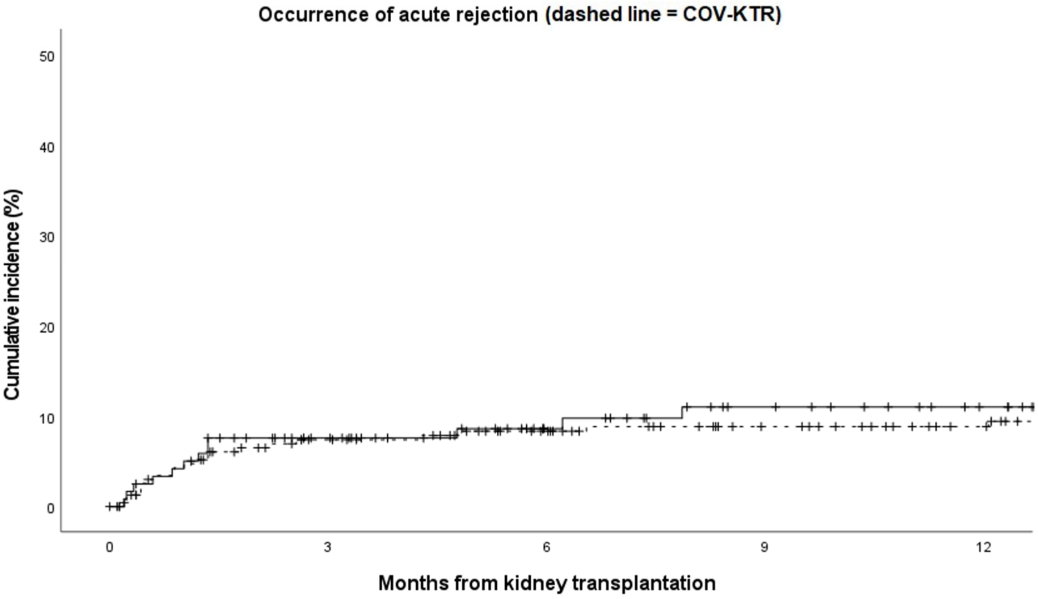

	Baseline (%)	3 months (%)	6 months (%)	12 months (%)
Pre-COV-KTR	0	8	9	11
COV-KTR	0	7	8	9
Number of patients exposed				
Pre-COV-KTR	122	115	89	64
COV-KTR	238	228	195	164

P = 0. 680
