# Supplementary material for: Outcomes of Patients Receiving a Kidney Transplant or Remaining on the Transplant Waiting List at the Epicentre of the COVID-19 Pandemic in Europe: An Observational Comparative Study
Source: Pathogens. 2022 Oct 3;11(10):1144. doi: 10.3390/pathogens11101144 (PMC9610233; doi:10.3390/pathogens11101144)
Supplement: Supplementary file 1 [file pathogens-11-01144-s001.zip › Supplementary Table S1.pdf]

Supplementary Table S1. Demographic and clinical characteristics of kidney transplant donors before (Pre-COV) or during (COV) the COVID-19 pandemic (Analysis A2).

Variables	Whole population (N = 360)	Pre-COV donors (N = 122)	COV donors (N = 238)	*p*
Donor sex (male)	202 (56.1)	68 (55.7)	134 (56.3)	1.000
Donor age (years)	55 (46–61)	54 (44–61)	55 (46–62)	0.510
Type of donor:				
DBD	253 (70.3)	82 (67.2)	171 (71.8)	0.394
DCD	32 (8.9)	13 (10.7)	19 (8.0)	0.436
ECD	143 (39.7)	48 (39.3)	95 (39.9)	1.000
LD	75 (20.8)	27 (22.1)	48 (20.2)	0.682
Donor risk factors:				
Cerebrovascular accident	143 (39.7)	46 (37.7)	97 (40.8)	0.649
Arterial hypertension	113 (31.4)	37 (30.3)	76 (31.9)	0.811
Last SCr >1.5 mg/dL	48 (13.3)	18 (14.8)	30 (12.6)	0.624
ICU admission	285 (79.2)	95 (77.9)	190 (79.8)	0.682
ICU stay (days)	3 (1–5)	3 (1–5)	3 (1–5)	0.948
Donor ethnicity:				
Caucasian	351 (97.5)	122 (100)	229 (96.2)	0.031
Afro-Caribbean	1 (0.3)	0 (0.0)	1 (0.4)	1.000
Other	8 (2.2)	0 (0.0)	8 (3.4)	0.055
Cold ischaemia time (minutes)	765 (630–960)	760 (630–940)	780 (620–965)	0.687

Abbreviations: DBD, donor after brain death; DCD, donor after circulatory death; ECD, expanded criteria donor; ICU, intensive care unit; IQR, interquartile range; KTR, kidney transplant recipient; LD, living donor.
