# Supplementary material for: Outcomes of Patients Receiving a Kidney Transplant or Remaining on the Transplant Waiting List at the Epicentre of the COVID-19 Pandemic in Europe: An Observational Comparative Study
Source: Pathogens. 2022 Oct 3;11(10):1144. doi: 10.3390/pathogens11101144 (PMC9610233; doi:10.3390/pathogens11101144)
Supplement: Supplementary file 1 [file pathogens-11-01144-s001.zip › Supplementary Table S2.pdf]

Supplementary Table S2. Demographic and clinical characteristics of kidney transplant recipients (KTR) before (Pre-COV-KTR) or during (COV-KTR) the COVID-19 pandemic: analysis A2.

Variables	Whole population (N = 360)	Pre-COV-KTR (N = 122)	COV-KTR (N = 238)	*p*
Recipient sex (male)	205 (56.9)	71 (58.2)	134 (56.3)	0.738
Recipient age (years)	52 (43–60)	52 (45–60)	51 (42–59)	0.366
Recipient ethnicity:				
Caucasian	324 (90.0)	111 (91.0)	213 (89.5)	0.714
Afro-Caribbean	7 (1.9)	1 (0.8)	6 (2.5)	0.430
Other	29 (8.1)	10 (8.2)	19 (8.0)	1.000
Renal replacement therapy	341 (94.7)	118 (96.7)	223 (93.7)	0.320
Haemodialysis	276/341 (80.9)	92/118 (78.0)	184/223 (82.5)	0.314
Dialysis vintage (months)	35 (16–57)	34 (16–62)	35 (16–56)	0.891
Primary kidney disease:				
Primary or secondary glomerulonephritis	173 (48.1)	56 (45.9)	107 (45.0)	0.911
Diabetic nephropathy	13 (3.6)	5 (4.1)	8 (3.4)	0.769
Polycystic kidney disease	66 (18.3)	25 (20.5)	41 (17.2)	0.473
Hypertensive nephrosclerosis	24 (6.7)	7 (5.7)	17 (7.1)	0.664
Tubulointerstitial disease	12 (3.3)	3 (2.5)	9 (3.8)	0.758
Genetic or congenital kidney disease	39 (10.8)	13 (10.7)	26 (10.9)	1.000
Uropathy	42 (11.7)	12 (9.8)	30 (12.6)	0.492
Thrombotic microangiopathy	24 (6.7)	7 (5.7)	17 (7.1)	0.664
Other	5 (1.4)	2 (1.6)	3 (1.3)	1.000
Pre-existing comorbidities:				
Arterial hypertension	311 (86.4)	106 (86.9)	205 (86.1)	1.000
Diabetes mellitus	43 (11.9)	19 (15.6)	24 (10.1)	0.169
Chronic obstructive pulmonary disease	49 (13.6)	19 (15.6)	30 (12.6)	0.516
Coronary artery disease	40 (11.1)	11 (9.0)	29 (12.2)	0.479
Obesity (BMI ≥30 kg/m^2)	27 (7.5)	10 (8.2)	17 (7.1)	0.833
CMV IgG positivity	312 (86.7)	109 (89.3)	203 (85.3)	0.328
EBV IgG positivity	312 (86.7)	107 (87.7)	205 (86.1)	0.745
HSV IgG positivity	286 (79.4)	96 (78.7)	190 (79.8)	0.785
VZV IgG positivity	348 (96.7)	118 (96.7)	230 (96.6)	1.000
HBV viremia	6 (1.7)	1 (0.8)	5 (2.1)	0.668
HCV viremia	4 (1.1)	1 (0.8)	3 (1.3)	1.000
Previous kidney transplantation	86 (23.9)	33 (27.0)	53 (22.3)	0.361
Last PRA (%)	0 (0–0)	0 (0–0)	0 (0–0)	0.510
N° Baseline DSA	0 (0–0)	0 (0–0)	0 (0–0)	0.440
Baseline DSA	23 (6.4)	10 (8.2)	13 (5.5)	0.364
HLA mismatch	4 (3–5)	4 (3–5)	4 (3–5)	0.345
Length of hospitalisation	13 (10–21)	15 (10–20)	13 (10–21)	0.950
ICU admission	79 (21.9)	26 (21.3)	53 (22.3)	0.893
ICU stay (days)	1 (1–2)	1 (1–4)	1 (1–1)	0.080
Induction immunosuppression:				
Anti-IL2R antibodies	179 (49.7)	56 (45.9)	123 (51.7)	0.318
rATG	204 (56.7)	75 (61.5)	129 (54.2)	0.217
Methylprednisolone	360 (100)	122 (100)	238 (100)	1.000
Anti-CD20 antibodies	24 (6.3)	7 (7.4)	15 (6.3)	0.824
Anti-C5 antibodies	34 (9.4)	7 (5.7)	27 (11.3)	0.090
Plasmapheresis	37 (10.3)	15 (12.3)	22 (9.2)	0.365
Polyclonal human immunoglobulins	31 (8.6)	13 (10.7)	18 (7.6)	0.327

Maintenance immunosuppression:				
Tacrolimus	357/357 (100)	120/120 (100)	237/237 (100)	1.000
MMF/MPA	347/357 (97.2)	116/120 (96.7)	231/237 (97.5)	0.738
Prednisone	349/357 (97.8)	117/120 (97.5)	232/237 (97.9)	1.000
Tacrolimus trough levels (ng/ml):				
After 1 month	9.1 (7.6–11.0)	9.1 (7.6–10.9)	9.1 (7.5–11.1)	0.773
After 3 months	8.8 (7.2–10.2)	9.1 (7.1–10.2)	8.4 (7.2–10.2)	0.586
After 6 months	8.5 (7.0–10.0)	8.9 (7.3–10.1)	8.4 (6.9–10.0)	0.295
After 9 months	7.9 (6.7–9.2)	7.7 (6.5–8.6)	7.9 (6.7–9.4)	0.372
After 12 months	7.9 (6.6–9.4)	8.1 (6.8–9.6)	7.7 (6.5–9.3)	0.501
Follow-up (months)	28.1 (13.2–37.8)	34.9 (27.6–41.5)	22.4 (10.4–43.6)	<0.001

Abbreviations: BMI, body mass index; CMV, cytomegalovirus; DSA, donor-specific antibody; EBV, Epstein-Barr virus; HBV, hepatitis B virus; HCV, hepatitis C virus; HLA, human leukocyte antigen; HSV, herpes simplex virus; IgG, immunoglobulin G; IL2R, interleukin-2 receptor; MMF/MPA, mycophenolate mofetil / mycophenolic acid; PRA, panel re-active antibody; rATG, rabbit anti-thymocyte globulin; VZV, varicella-zoster virus
