# Supplementary material for: Outcomes of Patients Receiving a Kidney Transplant or Remaining on the Transplant Waiting List at the Epicentre of the COVID-19 Pandemic in Europe: An Observational Comparative Study
Source: Pathogens. 2022 Oct 3;11(10):1144. doi: 10.3390/pathogens11101144 (PMC9610233; doi:10.3390/pathogens11101144)
Supplement: Supplementary file 1 [file pathogens-11-01144-s001.zip › Supplementary Table S3.pdf]

Supplementary Table S3. 1-year kidney transplant-related outcomes before (Pre-COV-KTR) or during (COV-KTR) the COVID-19 pandemic (Analysis A2).

Variables	Whole population (N = 360)	Pre-COV-KTR (N = 122)	COV-KTR (N = 238)	*p*
PNF	4 (1.1)	2 (1.6)	2 (0.8)	0.607
DGF	88 (24.4)	27 (22.1)	61 (25.6)	0.518
DGF duration (days)	7 (4–11)	7 (5–11)	7 (4–12)	0.761
CCI	23 (9–42)	23 (9–42)	23 (6–42)	0.472
SCr at discharge	1.42 (1.10–1.85)	1.34 (1.10–1.73)	1.46 (1.12–1.91)	0.103
SCr after 1 month	1.45 (1.15–1.81)	1.32 (1.07–1.63)	1.50 (1.17–1.88)	0.014
SCr after 3 months	1.40 (1.15–1.70)	1.33 (1.11–1.60)	1.43 (1.16–1.82)	0.017
SCr after 6 months	1.40 (1.16–1.70)	1.32 (1.15–1.60)	1.44 (1.17–1.79)	0.031
SCr after 9 months	1.39 (1.11–1.69)	1.35 (1.07–1.56)	1.41 (1.12–1.78)	0.085
SCr after 12 months	1.35 (1.10–1.64)	1.30 (1.06–1.57)	1.38 (1.12–1.71)	0.128
N° of hospital re-admissions	0 (0–1)	0 (0–1)	0 (0–1)	0.922
SARS-CoV-2 cases	42 (11.7)	0 (0.0)	42 (17.6)	<0.001

Abbreviations: CCI, comprehensive complication index; DGF, delayed graft function; PNF, primary non-function; SCr, serum creatinine.
