# Supplementary material for: Outcomes of Patients Receiving a Kidney Transplant or Remaining on the Transplant Waiting List at the Epicentre of the COVID-19 Pandemic in Europe: An Observational Comparative Study
Source: Pathogens. 2022 Oct 3;11(10):1144. doi: 10.3390/pathogens11101144 (PMC9610233; doi:10.3390/pathogens11101144)
Supplement: Supplementary file 1 [file pathogens-11-01144-s001.zip › Supplementary Table S4.pdf]

Supplementary Table S4. characteristics of kidney transplant recipients with SARS-CoV-2 infection.

Patient	Sex	Age	Primary kidney Disease	Pre-Existing Conditions					Induction Immunosuppression						
				HT	DM	COP D	CAD	BMI > 30	Anti-IL2R Ab	rATG	MP	Anti-CD20 Ab	Anti-C5 Ab	PEX	IVIg
1	M	54	PUV	No	No	No	No	No	No	Yes	Yes	No	No	No	No
2	F	51	Primary GN	Yes	Yes	No	No	No	No	Yes	Yes	No	No	Yes	Yes
3	M	44	ADPKD + Alport S.	Yes	No	No	No	No	No	Yes	Yes	No	No	No	No
4	M	61	Primary GN	Yes	Yes	No	Yes	No	No	Yes	Yes	No	No	No	No
5	F	52	Primary GN	Yes	No	No	Yes	No	No	Yes	Yes	No	No	Yes	No
6	M	38	ADPKD	Yes	No	No	No	No	Yes	No	Yes	No	No	No	No
7	M	61	HT + PRCC	Yes	No	No	No	No	Yes	No	Yes	Yes	No	Yes	Yes
8	F	46	Primary GN	No	Yes	No	No	No	Yes	No	Yes	No	No	No	No
9	M	61	Primary GN	Yes	No	No	No	No	No	Yes	Yes	No	No	No	No
10	M	34	Renal hypoplasia	Yes	No	No	No	No	No	Yes	Yes	Yes	No	Yes	Yes
11	M	66	Primary GN	Yes	No	No	No	Yes	Yes	No	Yes	No	No	No	No
12	M	76	Primary GN	Yes	No	No	No	No	No	Yes	Yes	No	No	No	No
13	M	22	Hypoplasia + VUR	No	No	No	No	No	Yes	No	Yes	No	No	No	No
14.	M	25	Primary GN	Yes	No	Yes	No	No	No	Yes	Yes	No	No	No	No
15	M	68	Primary GN	Yes	Yes	No	No	Yes	No	Yes	Yes	Yes	No	Yes	Yes
16	M	52	Primary GN	Yes	No	No	No	No	Yes	No	Yes	No	No	No	No
17	F	28	Secondary GN (SLE)	Yes	No	No	No	No	Yes	No	Yes	No	No	No	No
18	F	50	Nephronophthisis	Yes	No	No	No	No	No	Yes	Yes	No	No	No	No
19	M	65	Secondary GN (ANCA)	Yes	No	Yes	No	No	No	Yes	Yes	No	No	No	No
20	M	66	HT	Yes	No	No	No	No	No	Yes	Yes	No	No	No	No
21	F	24	Primary GN	Yes	No	No	No	No	Yes	No	Yes	No	No	No	No
22	M	28	Primary GN	No	No	No	No	No	Yes	No	Yes	No	No	No	No
23	F	45	Primary GN	Yes	No	No	No	No	No	Yes	Yes	No	No	No	No
24	M	61	Primary GN	No	No	No	Yes	No	No	Yes	Yes	No	No	Yes	No
25	F	27	aHUS	No	No	No	No	No	Yes	No	Yes	No	Yes	No	No
26	M	76	Nephronophthisis	Yes	No	No	No	No	No	Yes	Yes	No	No	No	No
27	M	29	VUR	Yes	No	No	No	No	Yes	No	Yes	No	No	No	No
28	M	30	Primary GN	No	No	No	No	No	Yes	No	Yes	No	No	No	No
29	M	48	VUR	Yes	No	No	No	No	Yes	No	Yes	No	No	No	No
30	M	55	Primary GN	Yes	No	No	No	No	Yes	No	Yes	No	No	No	No

31	M	52	Primary GN	Yes	No	No	Yes	No	Yes	No	Yes	No	No	No	No
32	M	49	Primary GN	Yes	No	No	No	No	No	Yes	Yes	No	No	No	No
33	M	57	Primary GN	Yes	No	No	No	No	No	Yes	Yes	No	No	No	No
34	M	22	Primary GN	Yes	No	No	No	No	No	Yes	Yes	No	No	No	No
35	M	39	HT	Yes	No	No	No	No	Yes	No	Yes	No	No	No	No
36	M	52	Alport S.	Yes	No	No	Yes	No	Yes	No	Yes	No	No	No	No
37	M	58	Primary GN	Yes	No	No	No	No	No	Yes	Yes	No	No	No	No
38	F	58	Primary GN	Yes	No	No	Yes	No	Yes	No	Yes	No	Yes	No	No
39	F	58	Primary GN	Yes	No	No	No	No	No	Yes	Yes	No	No	No	No
40	M	61	ADPKD	Yes	No	No	No	No	Yes	No	Yes	No	No	No	No
41	M	21	Denis Drash S.	No	No	No	No	No	Yes	No	Yes	No	No	No	No

Abbreviations: ADPKD, Autosomal dominant polycystic kidney disease; aHUS, atypical hemolytic uremic syndrome; BMI, body mass index; CAD, coronary artery disease; COPD, chronic obstructive pulmonary disease; DM, diabetes mellitus; EVIg, intravenous Immunoglobulin; GN, glomerulonephritis; HT, hypertension; IL2R, interleukin-2 receptor; PEX, plasma exchange; PRCC, papillary renal cell carcinoma; PUV, posterior urethral valves; rATG, rabbit anti-thymocyte globulins; S., syndrome; SLE, systemic lupus erythematosus; VUR, vesicoureteral reflux.
